# The Manchester guidelines for contralateral risk-reducing mastectomy

**DOI:** 10.1186/s12957-015-0638-y

**Published:** 2015-08-07

**Authors:** Narendra Nath Basu, G L Ross, D G Evans, L Barr

**Affiliations:** Nightingale and Genesis Prevention Centre, University Hospital South Manchester, Southmoor Road, Manchester, M23 9LT UK; The Institute of Cancer Sciences, The University of Manchester, Oxford Road, Manchester, M13 9PL UK; St. Mary’s Hospital, Oxford Road, Manchester, M13 9WL UK; Queen Elizabeth Hospital, Birmingham, B15 2TH UK

**Keywords:** Contralateral, Breast cancer, Risk-reducing mastectomy, Guidelines, Multi-disciplinary team

## Abstract

**Background:**

Rates of contralateral risk-reducing mastectomy (CRRM) are rising, despite a decreasing global incidence of contralateral breast cancer. Reasons for requesting this procedure are complex, and we have previously shown a variable practice amongst breast and plastic surgeons in England. We propose a protocol, based on a published systematic review, a national UK survey and the Manchester experience of CRRM.

**Methods:**

We reviewed the literature for risk factors for contralateral breast cancer and have devised a 5-step process that includes history taking, calculating contralateral breast cancer risk, cooling off period/counselling, multi-disciplinary assessment and consent. Members of the multi-disciplinary team included the breast surgeon, plastic surgeon and geneticist, who formulated guidelines.

**Results:**

A simple formula to calculate the life-time risk of contralateral breast cancer has been devised. This allows stratification of breast cancer patients into different risk-groups: low, above average, moderate and high risk. Recommendations vary according to different risk groups.

**Conclusion:**

These guidelines are a useful tool for clinicians counselling women requesting CRRM. Risk assessment is mandatory in this group of patients, and our formula allows evidence-based recommendations to be made.

## Background

The last decade has seen a marked increase in the numbers of women requesting contralateral risk-reducing mastectomy (CRRM) following a diagnosis of unilateral breast cancer [1]. This is despite a decreasing incidence globally of contralateral breast cancer (CBC) as a result of successful adjuvant therapies [2]. A contributing factor has been the introduction of genetic testing to routine clinical practice, but this only accounts for a small proportion of patients requesting the surgery. In breast cancer patients with a known *BRCA1/BRCA2* mutation, CRRM is associated with a 48–63 % [3, 4] survival advantage. Given that rates of contralateral breast cancer in this group are up to 4× greater than non-mutation carriers, a discussion of CRRM is easy to justify. For the majority of women with no known mutation, there appears to be little if any survival advantage to CRRM [5].

There is a wealth of literature on the appropriate management of women requesting bilateral risk-reducing mastectomy (BRRM) because of family history or known genetic mutation [5]. Interest in risk-reducing surgery has significantly increased recently, particularly since celebrities disclosed their experience of risk-reducing mastectomies. Existing guidelines on BRRM include the updated 2013 NICE Guidelines on Familial Breast Cancer in the UK [6]. Protocols exist for the counselling process for these women before embarking on the surgery, with the Manchester guidelines for BRRM being amongst the first [7]. However, no such protocols or guidelines are in place for women requesting CRRM after a diagnosis of cancer. The purpose of this paper is to propose a plan of management with regard to CRRM. Our protocol is based on a previously published systematic review of risk factors for contralateral breast cancer [8], a national survey of UK practice [9] and the Manchester experience of CRRM [10]. This is in a similar manner and for similar reasons to the published Manchester guidelines [7] for BRRM.

## Methods

### Assessment of the patient requesting CRRM

These guidelines have been formulated to aid clinicians dealing with requests for CRRM. Where possible, a level of evidence has been assigned from the designations set by the Centre of Evidence-Based Medicine. There are several steps in the process of preoperative assessment and counselling that are clinically important before an informed consent to CRRM can be given. These can be summarised as follows:

Step 1 Taking a history
Step 2 Calculating the risk of contralateral breast cancer
Step 3 Cooling off period whenever possible
Step 4 Multi-disciplinary team (MDT) discussion
Step 5 Patient consent

### Step 1: Taking a history

The first element of history taking is to determine the reasons behind a patient’s request to discuss CRRM. For the majority of women, the decision to request contralateral surgery is based on factors other than inherited genetic risk [11]. Women with breast cancer may have complex, multi-factorial reasons for requesting CRRM, and so the history should typically begin with open-ended questions to let the patient discuss her reasoning, objectives, hopes and fears. Objective assessment of this is challenging, with only a few reports in the literature [11].

Table 1 lists the main reasons patients request CRRM [12] and is verified by our own study of clinical practice in England [9]. Patients list fear of a second diagnosis, fear of chemotherapy and anxiety about their children’s future as the main drivers, followed by gene mutation status and family history—whereas surgeons rank gene mutation and family history as the main reasons to offer CRRM [9].

**Table 1:** Reasons patients request CRRM [9–12]

Reasons patients request CRRM
Gene mutation
Family history
Fear/anxiety—second breast cancer —long term survival
Avoid chemotherapy
Symmetry of reconstruction
Mistrust of surveillance
Offered TRAM/DIEP—a “once only” option

The breast cancer patient requesting CRRM is different to the patient considering BRRM. Although the latter may have experience of a family member’s breast cancer journey, they would not have had the personal experience of breast cancer, and their reasons for choosing risk-reducing mastectomy may vary significantly [11, 13, 14]. Fear of developing another breast cancer is a frequently expressed concern, but not necessarily related to whether or not this would influence life expectancy [15]. For some women, fear of having repeated chemotherapy is their main concern. For others, it is mistrust of annual mammographic surveillance particularly if their first breast cancer was mammographically occult, or if they have had a stressful ‘recall’ following a surveillance mammogram. Greater confidence in good outcomes following breast reconstruction may prompt a discussion of whether better symmetry might be obtained by bilateral rather than unilateral mastectomy, a factor that may also explain the ‘Angelina Jolie’ effect of increased interest in BRRM [16]. For women whose primary motive is better long-term survival, a simple explanation that CRRM will not achieve this in those who do not carry a gene mutation may stop the discussion going further. However, women may have strongly held motivations for CRRM quite independent of any effect on survival chances, and these need to be understood and recorded.

The clinical history also needs to assess the index breast cancer and highlight any potential poor prognostic indicators. It may be useful to objectively assess this risk using one of several validated predictive tools readily available. In addition, any co-morbidities should be identified that would influence further surgery and in particular reconstructive surgery. In step 5 of the process, a more detailed discussion is required of their expectations around breast reconstruction including a discussion of the risks and benefits of the reconstructive options in their case.

### Step 2: Calculating the risk of contralateral cancer

Women being considered for CRRM should have an objective assessment made of their risk of developing CBC, as well as an explanation of whether CRRM would or would not influence survival prospects. It is well documented that many women overestimate their personal risk of CBC and the survival benefit of CRRM and at the same time underestimate the adverse effects of the additional surgery [15, 17]. A significant proportion of breast cancer patients will undergo CRRM despite knowing there is no survival advantage in non-mutation carriers [17] with only a small proportion understanding the limited survival benefit in non-mutation carriers [17].

CBC risk is multi-factorial as seen in Table 2. It is important to calculate an individualised lifetime risk of CBC to stratify patients into different risk-groups, prior to consent for CRRM. This will facilitate the counselling process and provide a useful standard so that practices throughout can be audited.

**Table 2:** Summary of risk factors for CBC and levels of evidence [8]

Family history—<45 years with a first-degree relative (RR 2.5) —<55 years with first degree relative (RR 1.5) —first degree relative with bilateral disease (RR 3.5) Level II evidence (Reiner AS JCO—2013) [18]
Gene mutation status—*BRCA1/2* mutation (RR4) Level II evidence (Metcalfe 2004 JCO; Evans 2013) [4, 25]
Chest radiotherapy for Hodgkin’s lymphoma—rate of CBC unknown
Young age at diagnosis—<30 years 0.5–1.3 % annual CBC rate Level II evidence (Nichols, Lacey JCO 2011) [2]
ER status—ER positive (reference point RR 1) —ER negative (RR 1.3) Level II evidence [26]
Anti-endocrine treatment (risk reduction), tamoxifen 50 % Aromatase inhibitor 70 % Level I evidence [27, 28]
DCIS—0.6 % annual CBC risk of DCIS and/or invasive carcinoma (RR 1.0) Level I evidence [21]
Lobular histology combined with family history (RR2.0)
Oophorectomy under 40 years (risk reduction) (RR0.5)
Early menopause <45 year (risk-reduction)—published as abstract [29]

The risk of CBC in patients with known *BRCA* gene mutations is approximately 2–3 % per annum, and likely higher in *TP53* mutation carriers [18]. The baseline risk of CBC in patients with no family history is approximately 0.5 % per annum [19]. The use of anti-endocrines is associated with a 50–70 % risk reduction, with a greater reduction in risk from aromatase inhibitors [8]. These baseline risks can be modified by certain factors listed in Table 2. Patients diagnosed with unilateral breast cancer requesting CRRM and who have a strong family history should ideally have formal assessment of risk and genetic testing carried out by a clinical geneticist. In other cases, a useful objective assessment of risk of CBC can be calculated as follows.

Life expectancy at birth for women varies within the UK (range 78.5 years—Glasgow to Purbeck 86.6 year—Source ONS) [20], and we have used 80 years as the average life expectancy. Thus, to obtain the number of years of CBC risk, we can simply use the following calculation:

$$ 80\mathrm{years}\ \hbox{--}\ \mathrm{patients}\ \mathrm{age}\ \left(\mathrm{years}\right) = \mathrm{Number}\ \mathrm{of}\ \mathrm{years}\ \mathrm{of}\ \mathrm{C}\mathrm{B}\mathrm{C}\ \mathrm{risk}. $$

We can then use the quoted annual incidence of CBC at 0.5 % per year as a guide to the background risk for CBC in women diagnosed with breast cancer. To obtain a life-time risk based on this, we can use the following calculation (this assumes good life expectancy from the ipsilateral primary and typically would only apply to stage 1 cancers):

$$ \mathrm{Number}\ \mathrm{of}\ \mathrm{years}\ \mathrm{of}\ \mathrm{C}\mathrm{B}\mathrm{C}\ \mathrm{risk} \times 0.5\% = \mathrm{Life}-\mathrm{time}\ \mathrm{risk}\ \mathrm{of}\ \mathrm{C}\mathrm{B}\mathrm{C} $$

Once this value has been calculated, we can modify the risk based on patient’s personal risk profile as follows:

- Estrogen receptor (ER)-positive disease and on anti-endocrine treatment—multiply by 0.5 (50 % risk reduction)
- Gene carriers—multiply by 4 (2 % annual incidence of CBC)
- Oophorectomy under 40 years (surgical, chemotherapy induced or natural)—multiply by 0.5 (50 % reduction)
- Family history—multiply by 2 (unpublished data based on our own family history clinic)

Note: where numerous factors are being considered, the multiplicative interaction of factors is not known. For those patients with a known genetic mutation and a family history, consider modifying risk based on gene mutations (i.e. multiply by 4) only. Anti-endocrine treatment in those who have had oophorectomy under the age of 40 years, consider risk reduction by one factor (i.e. multiply by 0.5) as there is no data to our knowledge on whether the relationship is additive.

The quoted baseline annual incidence of CBC of 0.5 % per annum is reduced in ER-positive tumours by 50 % after 5 years of adjuvant tamoxifen but even more by an aromatase inhibitor.

Unilateral ductal carcinoma in situ (DCIS) is associated with an increased risk of developing a contralateral invasive cancer or DCIS. This annual risk is estimated at 0.6 % [21] and may be used in the above formula.

These calculations are of course only a guide, but they are useful to stratify risk into clinically relevant risk categories such as the following:

- Low risk
  <10 % remaining life-time risk of CBC
- Above average risk
  10–20 % remaining life-time risk of CBC
- Moderate risk
  20–30 % remaining life-time risk of CBC
- High risk
  >30 % remaining life-time risk of CBC

The use of risk categories has proved invaluable in the context of BRRM, and there are several validated tools now to calculate breast cancer risk based on family history and lifestyle factors for women without a cancer diagnosis [22] (i.e. Manchester Score, Tyrer-Cuzick, BOADICEA). At present, there are no validated tools to calculate CBC risk in the context of CRRM. Our experience of using the above method has been previously presented in abstract form and is a useful clinical tool for validation in future studies [10].

### Step 3: Cooling off period whenever possible

The decision-making process around BRRM is characterised by several months of pre- and post-test counselling. In contrast, women who request CRRM may do so within a few hours or days from their diagnosis of breast cancer. Nationally defined targets for prompt treatment following a diagnosis of breast cancer may impact on the shared decision-making process by limiting the time available for careful consideration of the pros and cons of CRRM.

For the majority of patients, it is probably in their best clinical interest to defer any decision about CRRM until after their primary cancer treatment has been completed. This “cooling off period” minimises the risk that they make a decision for CRRM as a knee-jerk reaction at a time when they are emotionally vulnerable. There are exceptions to this recommendation however. These include the patient with a known BRCA mutation who may have made a decision many months or years previously to undergo bilateral mastectomies for therapeutic and risk-reducing reasons in the event of a cancer being diagnosed. Non-mutation carriers in the high-risk group defined in step 2, such as those with a significant family history or previous mantle radiotherapy, may also decide on CRRM as part of their primary treatment on diagnosis of a unilateral cancer and again may not benefit from a cooling off period.

Overall, the majority of women are satisfied with their decision of CRRM up to a decade following surgery [Ref. Frost et al., JCO 2005]. However, it is not known whether timing of this decision impacts on the level of satisfaction. Where feasible, this step allows women to carefully consider the various options available to them in a non-time constrained manner. However, this is not always possible as the need to treat the affected side and possible reconstructive options will influence the decision-making process and the speed at which happens.

Patients considering an immediate transverse rectus abdominus muscle/deep inferior epigastric artery perforator (TRAM/DIEP) flap reconstruction for their primary therapeutic mastectomy are also an important possible exception and are discussed further in step 5.

### Step 4: MDT discussion

Given the complexity of the decision-making process, all women considering CRRM should be assessed in a multi-disciplinary setting—surprisingly, this is not universally practised in the UK [9]. The core members of the team should include breast care nurse, breast surgeon, oncologist, radiologist and pathologist and where possible, an oncoplastic-reconstructive surgeon familiar with the various reconstructive options including free TRAM/DIEP. For patients with a family history, discussion with a clinical geneticist of their risk of CBC and the possibility of genetic testing should be offered during the patients cooling off period. Often, the breast care nurse will have developed a close relationship with the patient and is the patient’s advocate at the MDT meeting. She should have the option of requesting additional psychology assessment if she feels necessary, but we would not regard this as mandatory. The patients’ reasons for requesting CRRM should be discussed at the MDT in the light of her objective risk of CBC, influence on survival chances, risks and benefits of the additional surgery, and the alternative options around surveillance and imaging. Additionally, it is useful to review the imaging, as some women may be particular challenging to offering radiological surveillance on the contralateral side.

The main benefit of a multi-disciplinary approach is that requests for CRRM can be scrutinised across various specialties and facilitate shared decision-decision making [23]. A recent report showed that introduction of this approach resulted in almost a third of requests for CRRM being declined, mainly due to a low risk of CBC in light of a high risk of systemic relapse. There is good evidence across various specialties supporting this form of collaborative working [24]. Our own approach enables the MDT process to consider each request for CRRM on an individual basis prior to making a consensus-led decision with the option of patient-led appeal for extenuating circumstances.

### Step 5: Patient consent

Each of the steps above is part of the consent process, commencing with a clear discussion of the benefits that the patient hopes to achieve with CRRM, and a clear explanation of the objective risk of CBC and whether or not any survival advantage can be achieved. Particularly for the non-gene carrier where no survival advantage exists, the conclusion that CRRM is appropriate and has the support of the MDT meeting should be recorded in the clinical notes. The next step is to explore the various reconstruction options available to the patient if that is what she wishes, combined with a clear explanation of the limitations of such surgery, its risks and complications. Unrealistic expectations of a perfect outcome and no postoperative complications should be addressed if present. The final step is the signing of a formal consent form, on which there is only sufficient space for the recording of a brief summary of the risks and benefits; all of these prior discussions should be recorded within the clinical notes prior to the actual signing of the form.

A particularly challenging situation is the patient who requests CRRM at the same time as her therapeutic mastectomy as part of her primary treatment. This puts the surgeon under time pressure to go through a complex consent process. For patients in the high-risk group defined in step 2, the situation may be fairly straightforward as often the patient will have had considered bilateral mastectomy as her preferred option long before the actual diagnosis. For patients undergoing primary neo-adjuvant systemic therapy, the situation is also more straightforward because a period of several months of “cooling off” between those first discussions and the final decision to proceed give the surgeon and his/her MDT time to complete the above steps. For the patient requiring a therapeutic mastectomy as her first treatment, and who chooses an immediate TRAM/DIEP flap as her preferred reconstruction, the decision has to be made within a few days, as otherwise the option of bilateral symmetrical reconstruction is lost forever. In these circumstances, there may be no time to proceed to a formal genetic assessment or to offer a cooling off period. The opportunity for CRRM should probably not be denied to the patient in those circumstances, as long as the risks and benefits are clearly explained and approved by the MDT, and recorded in the clinical notes.

Currently, there is not enough provision in the UK for every MDT to have an oncoplastic surgeon who can offer free TRAM/DIEP recon as a core member. Many MDTs in the UK have to refer out with to an extended MDT member (usually a plastic surgeon) which causes time delays and lengthens the decision-making process for patients and anxiety levels. Ideally, all MDTs should include an oncoplastic surgeon familiar with the various reconstructive options.

Women who are deemed not suitable for CRRM are currently offered annual mammography surveillance for 5 years or up to the age of 50 years—whichever is longer.

## Discussion

The management of breast cancer patients requesting CRRM is complex and best undertaken in a multi-disciplinary setting. Several challenges exist for clinicians in this setting. The first is their inability to calculate accurately the individualised risk of CBC for each patient given the numerous risk factors already described. We have suggested a potential framework, but development of a validated algorithm remains a high priority in research in this area of study. The other challenge remains managing the expectations of women for whom CRRM offers no survival advantage and who form the vast majority of women requesting CRRM.

This protocol serves as a tool for clinicians involved in this complex shared decision-making process with their patients. The simple risk calculation that we have described is based on a published systematic review of known risk factors and provides a clinically useful method of assigning patients to a variety of risk categories. It is hoped that this risk calculation will aid both patients and clinicians to jointly come to a decision regarding CRRM following a clear explanation of the objective risks of CBC and the potential benefits, risks and limitations of CRRM. We acknowledge that it is not possible to be prescriptive in terms of who should be allowed or refused CRRM.

The main limitations of this protocol are that recommendations are based on a systematic review of known risk factors which have not been assessed in a clinical setting. As such, the estimates of contralateral breast cancer risk need to be used with caution when multiple factors are being considered given that there is a limited evidence base to assess if this interaction is multiplicative. This formula has been designed to act as a guide rather than a precise tool for the objective assessment of risk. Efforts are underway to validate this protocol in a large retrospective study. However, to our knowledge, there are no established guidelines to aid the clinician to objectively assess requests for CRRM. As such, this protocol is the first to attempt to address this important issue. We hope that in the interim, these guidelines and the steps described within them help in reaching a shared decision. This will ensure each request for CRRM is judged based on its own merits.

### Ethical approval

The authors confirm that this manuscript conforms to ethical standards as set by the Committee on Publication Ethics (COPE).

## Abbreviations

CRRM: Contralateral risk-reducing mastectomy
CBC: Contralateral breast cancer
BRRM: Bilateral risk-reducing mastectomy
MDT: Multi-disciplinary team
TRAM: Transverse rectus abdominus muscle flap
DIEP: Deep inferior epigastric artery perforator flap
DCIS: Ductal carcinoma in situ
